# Factors associated with staying or leaving a dentist leader’s position – a qualitative study

**DOI:** 10.1186/s12903-016-0206-z

**Published:** 2016-04-16

**Authors:** Tiina Tuononen, Anna Liisa Suominen, Johanna Lammintakanen

**Affiliations:** University of Eastern Finland, Kuopio, Finland; Department of Oral and Maxillofacial Surgery, Kuopio University Hospital, Kuopio, Finland

**Keywords:** Dentists, Leadership, Personnel turnover, Qualitative research

## Abstract

**Background:**

Leadership and leaders have important roles today, possibly even more so in the future, since major organizational changes will occur throughout the health care sector. Tomorrow’s leaders will need to be competent and motivated. It is important to clarify the factors why some individuals stay and some quit leadership positions. We investigated factors associating with dentist leaders’ likelihood to stay in or leave a leadership position.

**Methods:**

Data were gathered while or after participants attended “the Special Competence in dental administration for leading dentists” education, utilizing the method of empathy-based stories. Participants wrote short essays on the basis of two contrasting frame stories, i.e. an imagined situation where either they left (Leavers, Group 1) or stayed in (Stayers, Group 2) a leadership position. Content analysis was used inductively to describe intent to stay or to leave factors and categorized according to the theory of “Career Anchors”.

**Results:**

The factors were not only specific to groups, since several common factors were also found such as satisfaction with leadership position even though the post was not initially the participant’s voluntary decision and the inadequate time for leadership work. Factors in both groups formed three themes: personal, working community, and health care sector levels. Both groups expected conditions to improve in their organizations, even though there were major concerns about on-going changes. Concurrently the uncertainty and the instability in the health care sector and the status of oral health care worried both groups. Leaver-specific factors were loneliness of leadership position, lack of support and the appropriate salary combined with the excessive number of duties. Stayer-specific factors were enthusiasm for leadership supported by education and possibility of develop oral health care as part of health care. The General Managerial Competence anchor was the dominant career anchor, especially among the Leaders.

**Conclusions:**

Working as a dentist leader is both demanding and challenging. In order to succeed and be personally satisfied and fulfilled in these leadership positions, it is essential to recognize either supporting or enervating factors towards leadership positions and that appropriate education, support, and time for leadership are needed.

## Background

Even though much research has been conducted into leadership in the nursing and medical professions, dentistry seems to be something of an exception; only a few career development or leadership studies have focussed on dentistry [1–4] or dentist leaders [5–7]. Kottonen [1] and Gallagher et al. [2] studied young dentists’ and dental students’ career choices, Gilmour et al. [3] assessed the career satisfaction of general dentists and Boshoff et al. [4] examined the job involvement and career orientation of different individuals working in the professions including dentists. Dentist leaders have been studied by Alestalo and Widström [5, 6] who examined the work and roles of dentist leaders during times of major organizational upheavals, and Bolin and Shulman [7] who studied employment-related factors for both dentists and dentist leaders i.e. retention, recruitment, work environment perceptions and salaries.

It is important to investigate factors which possibly influence on the decisions made by dentist leaders if we are to understand why some become frustrated and leave whereas others thrive after being appointed to a leadership position. Previously reported factors to influence on work dissatisfaction or turnover intents among dentists were: lack of professional autonomy, stress, inadequate financial compensation and in particular for individuals in leadership positions - inadequate time for administration duties [3, 7–9]. Conversely, positively associated factors for job satisfaction were overall professional satisfaction, income, respect and work time control [3]. Other factors supporting retention were the length of service and the freedom to exercise professional judgement; common factors found among both dentists and dentist leaders [7]. Alestalo and Widström concluded that the lead dentists had higher job satisfaction when they had applied for the leadership positions by themselves, when they felt that they had received sufficient leadership education, and when they worked in larger public dental service (PDS) units [6].

We considered that it would be interesting to apply career development theory as a novel way of assessing career choices, in particular to determine which so-called career anchors [10, 11] could explain dentist leaders’ career choices i.e. whether they would be likely to remain in or leave a leadership position. Schein [10, 11] stated that the career anchors are formed of three basic components which make up an individual’s professional self-image: self-experience of their own competence, motives and needs; and attitudes and values. Furthermore, Schein [11] postulated that a career anchor is a value which an individual is not willing to give up under any circumstances and therefore it could be either the retention or turnover factor. Although anchors (Table 1) seem to be rather permanent, in fact they can change with time as the individual gains new experiences. There is only one published article that has applied career anchors to dentists; this was from South Africa and it examined job involvement and career orientation in 14 professions [4]. Two career anchors called “Technical and Functional Competence” and “Pure Challenge” were found to be the most predictive of job involvement among dentists. However, there do not seem to be any studies which have utilized the concept of career anchors to examine the dentist leaders.

**Table 1:** Career anchor descriptions [20]

Technical and Functional Competence	This kind of person likes being good at something and will work to become a guru or expert. They like to be challenged and then use their skills to meet the challenge, doing the job properly and better than almost anyone else.
General Managerial Competence	These people want to be managers. They like problem-solving and dealing with other people. They thrive on responsibility. To be successful, they also need emotional competence.
Autonomy and Independence	These people have a primary need to work under their own rules and ‘steam’. They avoid standards and prefer to work alone.
Security and Stability	These people seek stability and continuity as a primary factor of their lives. They avoid risk and are generally ‘lifers’ in their job.
Entrepreneurial Creativity	These people like to invent things, be creative and most of all to run their own businesses They differ from those who seek autonomy in that they will share the workload. They find ownership very important. They get easily bored. Wealth, for them, is a sign of success.
Service and Dedication to a Cause	Service-orientated people are driven more by how they can help other people than by using their talents. They may work in public services or in areas such as human resources.
Pure Challenge	People driven by challenge seek constant stimulation and difficult problems that they can tackle. Such people will change jobs when the current one gets boring, and their career can be varied.
Lifestyle	Those who are focused first on lifestyle look at their whole pattern of living. Rather than balance work and life, they are more likely to integrate the two. They may even take long periods of time off work in which to indulge in passions such as travelling.

Competent leadership from motivated leaders are important today and will become even more crucial in the future. Deficits in both human and financial resources along with the ageing population and different organizational reforms have exerted a significant impact on the leadership in all areas of the health care sector including oral health care. It is important to clarify the factors which motivate individuals to seek and stay in the leadership positions as well as factors which have influence on their turnover decisions.

## Methods

The material for this study was collected in the spring 2013 in two different settings by using the method of empathy-based stories - also known as passive role-playing stories or non-active role-playing - [12, 13] where the study participants are asked to write a short imaginary story or essay on the basis of the provided frame stories. This method has been previously used especially in sociology, social psychology and pedagogy [12, 14–16]. The method of empathy-based stories is a variation of the non-active role-playing method [17, 18] and it was originally developed in social psychology to elucidate an individual’s personal interpretations of different scenarios [12, 13, 18]. The key point in this method is that there should be at least two different versions of the frame story but with one major difference. Essays can be based on the participants’ own experiences or they can be written on what they imagine would be the case i.e. they do not need to be based on reality, simply they should contain aspects that the respondent feels would be relevant.

In our study, the basis frame story asked the participants to peer 5 years into their imaginary future when they would be a dentist leader i.e. the target year was 2018. The participants (*n* = 25) (Table 2) were divided into two groups giving them one of two different frame stories; in the first they imagined the situation in which they were planning to leave the leadership position (Group 1: Leavers, *n* = 13) in the second scenario, they were asked to envisage the situation where they remained in their current imaginary leadership position (Group 2: Stayers, *n* = 12). The participants were unaware that there were two alternative scenarios, they wrote only one essay on the outcome which they had been randomly allocated. The participants were requested to consider what factors would have influenced the imaginary situation, what were their expectations and achievements, and how they planned to go on in their imaginary career from 2018 (Table 3). Furthermore, the study participants were asked some background questions of their experience as a dentist or as a dentist leader and whether they were working in the public or private sectors. The study participants provided informed consent while participating voluntarily in the study and they were told that their anonymity would be guaranteed in all phases of the study.

**Table 2:** Study participants and their background characteristics

Study attendees	n	Dentist experience years mean (range)	Leadership experience years mean (range)	Working sector
Total	25	20 (3–32)	2.5 (0–10)	20 public
				2 private
				3 both
Group 1, Leavers	13	20 (3–32)	2.5 (0–7)	11 public
				2 both
Group 2, Stayers	12	20 (4–31)	2.5 (0–10)	9 public
				2 private
				1 both

**Table 3:** The two frame stories with the differences in their outcomes which were used in the study

Group 1 - Leavers:	You have attended/You are attending the education of the Special competence in dental administration for leading dentists. We would like you to imagine your situation in five years’ time – in the year 2018. You are employed in a leadership position in a dental health unit but you are seriously considering resigning from your job. Can you write about the reasons why you have come to this decision? Please tell about the factors that have influenced this decision? How did you end up in this situation? What were your expectations from the position and what did not work out as expected? What do you think that the future from 2018 onwards holds for you?
Group 2 –Stayers:	You have attended/You are attending the education of the Special competence in dental administration for leading dentists. We would like you to imagine your situation in five years’ time – in the year 2018. You are employed in a leadership position in a dental health unit and you are really satisfied with your situation. Can you write about the factors which have led to this satisfactory situation? How did you end up in this situation? What were your expectations from the position and what have actually come true? What do you think that the future from 2018 onwards holds for you?

The first data collection was implemented in April 2013 to test the study arrangement with a group of dentists (*n* = 6) who had previously completed the programme of the Special competence in dental administration for leading dentists (Special Competence). They were selected on the basis of experience and willingness to take part in the study. All of the initially invited dentists participated in the study. The collection of the remainder of the material took place in a two-day course organized in May 2013 as part of Special Competence education 2012–2013. All of the participants attending that course (*n* = 19) volunteered to take part in the study.

In the first data collection, the written guidance was provided by e-mail. Guidance included the request that the essay should be written independently and promptly after reading the instructions and its completion should not take more time than 20 min. The respondents returned their typed essays by e-mail immediately after completing the task. The second data collection took place in a meeting room at the beginning of the second day of Special Competence course. In this instance, the first author (TT) spoke about the task and answered any questions and then the respondents started to compose write their own hand-written essays, without any discussion with fellow course members; in this occasion, the respondents were told that they should not spend more than 20 min writing their essays after they had received the oral instructions.

Since both frame stories were identical and the material collecting mode did not substantially change, we could combine the essays from two different settings in this analysis.

**Fig. 1:**
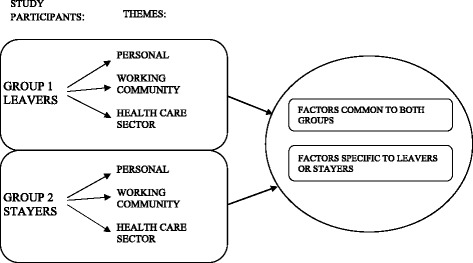
Schematic representation of the inductive content analysis conducted in this study in the identification of intent-to-leave or intent-to-stay characteristics

We used the content analysis method [19] to analyze the data in two different ways: A) inductively to describe intent to stay or leave factors and B) according to the career anchor theory. The materials in both groups (Leavers and Stayers) were subjected to inductive content analysis to assess the contrasting aspects and factors either supporting leaving or staying in the leadership position. During this part of the study, three distinctive levels became apparent; 1) the personal level, 2) the working community level, and 3) the health care sector level were found to be the main theme categories in both groups. These themes formed the basis for ascribing the sentiments identified as intent-to-leave or intent-to-stay and in addition the factors which were either common in both the groups (Stayers and Leavers) or specific factors either to the Leaver or Stayer group were identified and quantified (Fig. 1).

The essays were then subjected to a second analysis, this time according to the theory of career anchors utilizing the descriptions provided by Schein [20, 21].

## Results

The participants had similar educational backgrounds since they all had degrees in dentistry. The groups were also similar with regard to experience. The mean experience as a dentist was 20 years (range 3–32) and as a dentist leader 2.5 years (range 0–10), respectively (Table 2).

Factors associated with leaving or staying in the leadership position were both common and group specific. Three main theme categories were found to describe the factors in both groups. Out of the eight possible career anchors, the “General Managerial Competence” anchor was most often found. There were differences in the types and frequencies of anchors mentioned in the essays of the two groups.

### Common factors by both: Leaders and Leavers

#### Personal level factors

Factors supporting retention were education and possibilities to learn about the working organization and oneself while in a leadership position. The satisfaction of the leadership position was described in a few essays even though the post had not initially been a goal or even voluntarily sought. The leadership position was estimated as representing a good alternative to full-time clinical work. The informants also emphasized that the leadership position offered greater freedom to organize one’s own work. There were a few common intent-to-leave factors, i.e. a high emphasis was placed on pursuing a clinical career in addition to lack of time to pursue the administration duties.

“I did not intentionally seek out this position, but I am very satisfied. One can see how different work experiences provide a sense of perspective. I consider it to be a benefit although previously I have not valued it as such.” (Stayer, Group 2)

“At present, I think it is the most unlikely that I will proceed to the position of chief dentist leader in the municipality. It is much more likely that I will move towards pursuing a clinical specialization.” (Leaver, Group 1)

#### Working community level factors

Common factors found in this level of the analysis included positive future views while expecting that conditions would improve in their organizations. At the same time, there were common major concerns about how on-going changes would affect their own organizations. The wish to develop and improve the function of the working community was a common goal.

“From 2018, things will get better as the numbers of qualified people will increase and this will relieve work-related stresses. In addition, the management work will also become easier as the personnel’s workload decreases.” (Stayer, Group 2)

#### Health care sector level factors

Uncertainty and instability i.e. expectations of budget cutbacks and organizational changes throughout the social and health care branches worried both the groups. The importance of oral health care as a significant factor in the health care was emphasized.

“Today there is uncertainty due to politically-motivated structural changes in the social and health care sector and one has to be ready for anything, and especially to be able to live with the continuing uncertainty while waiting for unpredictable future changes.” (Stayer, Group 2)

### Group specific factors for either Leavers or Stayers

#### Personal level factors

With respect to the Leaver group, many factors were mentioned to explain why they were contemplating quitting i.e. stress, the lack of an appropriate salary, no real power to make meaningful decisions, the excessive number of the duties as well as the loneliness and the lack of support in the leadership position. Other factors mentioned were the undesirable change in the work concept forced on the respondent by the upper echelons of health care administration, the ageing of the individual, and a long working career.

“Too many burdens are loaded onto the shoulders of the dentist leader and there is not enough time. It seems that every municipality suffers from too little financial resources and too many patients.” (Leaver, Group 1)

The Stayer group mentioned fewer specific personal level factors. The enthusiasm for leadership was supported by receiving the appropriate education and the real impact possibilities. The satisfaction gained while solving the difficult issues and possibilities to create something innovative by themselves were mentioned as supporting staying.

“How did I arrive at this situation? Initially, there was some kind of vision about how I wanted to tackle my tasks and what types of goals I would set for myself and the unit I direct; subsequently these plans have been achieved, at least to some extent. Even when things did not turn out as I had hoped and planned, by adopting an open approach and trusting in others, it has been possible to reach a functioning outcome.” (Stayer, Group 2)

#### Working community level factors

The Leaver group emphasized staff related factors with the major concern being the lack of qualified staff and the ageing of the personnel as well as the dissatisfaction experienced by the personnel. In addition to human resources, the financial resources and the quality of the treatments being provided were also the factors which were mentioned.

“Just now it seems that all the juices are being squeezed out of the general dentists, with more and more clinical work being needed. The numbers of patients requiring treatment increase all the time but there is no recruitment of new staff or enlargement of premises to meet the demand.” (Leaver, Group 1)

The participants who envisaged staying mentioned one specific factor not stated by the Leavers, i.e. the concept of a leader with a positive attitude as the creator of a positive working community. The vision of the desired working community was described in one essay:

“All of my employees are not personal friends, but I have complete faith in their professional competence. Since I trust their actions I am ready to defend them to the very end. We may be different personalities but we take care of our professional work with care and diligence. All of the employees seem to enjoy their work and get satisfaction from doing it well. In this way, work does not simply consume personal resources but instead it is rewarding for the worker. Then work is what it is supposed to be – a part of your life. But only a part, not every aspect of life itself.” (Stayer, Group 2)

#### Health care sector level factors

The Leaver group had one specific concern; a worry about the structure and the quality of the oral health care. The chief dental officers should be at the highest level and have an equivalent status as the health care chief officers, plus they need to feel that they have possibilities to influence their units work in expanded areas in the future.

“The limited resources present in the health services may well be one reason why I am considering quitting. For example, the financial budget is so small that it is impossible to provide satisfactory services or there may be shortages of qualified personnel. Furthermore, another reason why I may decide to quit would be that there was some part of the upper executive management class whom I felt did not trust my capabilities at all.” (Leaver, Group 1)

There were some factors which were specific to the Stayers i.e. the concept of perceiving a wider perspective of the leadership position as well as the possibility to develop the oral health care as a part of the health care were described.

“I have always wanted to be a provider of dental health services, but also taking into account the resources and needs. In this respect, I have undoubtedly succeeded.” (Stayer, Group 2)

### Career anchor feature findings

#### “General Managerial Competence” anchor was found to be the dominant career anchor

There are eight possible career anchors (Table 1), of these, three were strongly emphasized, three were less featured, and two were not mentioned at all. In both groups, “General Managerial Competence” emerged as the strongest anchor, especially in the Stayer group. The desire to be able to integrate the efforts of others across functions and to be in charge and responsible for the output of one’s own unit, were mentioned in a few essays. The essays of the Leaver group more often displayed the features of the “Technical and Functional Competence” anchor. Many participants were planning further education and were more clinically orientated even though they still were in a leadership position. The features of the third emerged anchor “Service and Dedication to a Course” were equally common in both groups. In many of the essays, the concerns about the future within the overall health and in particular, the oral health sectors as well as the possibilities to ensure the supply of high quality services emerged. In the Leaver group, a few career anchor features were linked to “Lifestyle” (wish to balance life between work and life outside work). Mentions were made to certain features such as “Autonomy and Independence” (wish to be able to decide about your own work) and “Entrepreneurial Creativity” (wish to create something new/special as a result of one’s own efforts) anchors were found, but no clear referrals to “Security and Stability” and “Pure Challenge” anchors were detected in any of the essays.

## Discussion

Both common and specific factors associated with staying or leaving in the leadership position were found and they could be divided into three main theme categories: personal, working community, and the health care sector levels. These three themes may relate to the three dimensions of the work done by dentist leaders: primarily they are clinicians although at the same time they occupy a leadership position, secondly they are leaders/managers in their working units and representatives of the employer to their subordinates and finally, they are representatives, even lobbyists, for the oral health care in the health care organizations. Intent-to-leave feelings were most often described in the Leaver group and intent-to-stay feelings in the Stayer group, which was to be expected on the basis of the study design, but many common factors were also found in the essays of both groups. Career anchor signs or features could be found in the essays and findings differed according to the group and the identified career anchor signs seemed to support both intent-to-leave and intent-to-stay factors.

### Common factors across both groups

A positive attitude towards leadership was found in the essays although all of the study participants had not themselves actually decided to pursue a leadership position. This contrasts with the findings of Alestalo and Widström [5] where the respondents were found to be more likely to be better motivated in their leadership work when they had applied for this kind of position. Even under the pressure of organizational reform, both groups shared a common goal to develop and improve the effectiveness of the working community from their leadership position. Similar topics were mentioned by Alestalo and Widström [5] as well as in the study of Ham et al. [22] where the chief executive doctors stated that they had better opportunities to achieve organizational and service improvements on a more extensive scale than was possible in clinical work.

In our study the possibility to arrange one’s own work more freely while in the leadership position was considered as important and leadership was viewed as representing a good alternative to full-time clinical work. On the other hand, the one common intent-to-leave factor was the intention to pursue a clinical career rather than adopting a leadership role, this was especially apparent in the younger participants. Additionally in both groups, the lack of time for leadership work was mentioned as in the study of Bolin and Shulman [7] which emphasized that appropriate amounts of time should be provided for administrative duties.

With respect to the health care sector level factors, one common future concern was related to uncertainty and instability. One emerging common point was the status and the importance of oral health in the health care sector. In fact, this has been a topic of intense debate throughout the dental profession in Finland in anticipation of the imminent social welfare and health care reform.

### Specific factors in the groups

Many Leavers mentioned factors reflecting their sense of insecurity or feelings that did not receive appropriate respect, such as the lack of support from others, the lack of authority, the feeling that compensation and reward were inadequate as well as the belief that too much was expected. This is reminiscent of the results of an interview study of chief executive doctors which highlighted the insecurities associated with being a chief executive [22]. The results of our study are also in agreement with earlier reports [3, 5, 6, 8, 9]. On the contrary, the remuneration was not associated significantly with the intent-to-leave in the study of Bolin and Shulman [7].

In the Stayer group, the enthusiasm for leadership was described as a reason for the intent-to-stay. Similar results were reported in the study of Alestalo and Widström [6] where the vast majority (87 %) of the dentists in leadership positions stated that they gained a considerable sense of reward for being a lead dentist.

### Career anchors

The “General Managerial Competence” anchor signs were the most common outcome especially in the Stayer group and this could describe the factor for the intent to remain in a leadership position. The second most common outcome anchor “Technical and Functional Competence” was found more in the Leaver group and it could account for their intention to resign from their leadership position. In the study of Boshoff et al. [4], “Technical and Functional Competence” was the second of the two main anchors predicting the job involvement of dentists. This may illustrate the difference between dentists and dentist leaders. In particular, the younger attendees wrote about their intent to undertake clinical specialist education. The interest in leadership seems to arise with time and experience. In a British study of dental students [23] over one-third (37 %) of the study participants, anticipated participating in leadership roles in dental associations. The other important anchor described in the study of Boshoff et al. [4] i.e. “Pure Challenge” was not found in this present study. Future research into the career anchors of the dentist leaders is clearly needed; this will require evaluation with more specialized methods and more experienced dentist leaders should be interviewed. An awareness of the career anchors relevant to dentist leaders could help an individual to apply for an appropriate position and at the same time to encourage organizations to be able to offer suitable positions or tasks to their leaders.

### Validity and assessment of the methods

The method of empathy based stories can be used as the supplement to the traditional survey methods; it differs from many other qualitative methods which can be used to generate data. It has been described as a powerful method for gaining rapid feedback and since the data collection is straightforward in most cases, it is very suitable in exploratory studies and it was chosen here as a relevant way of asking the respondents to think about their future [13, 16]. As far as we are aware, this is the first time that this method has been used to study dental professionals.

Ethical issues were taken into account since all of the participants were told that participation was purely voluntary and they provided their consent for responses to be utilized for research purposes. The study informers wrote their own stories on the basis of the frame story where they imagined their possible future according to its basic premise. The variation in the frame stories seemed to function rather well. Only a few essays were somewhat superficial and concerned with general matters. The background of the first author (TT), i.e. she is a dentist and thus a colleague and she has also worked in a leadership position, was kept in mind and taken into consideration in all phases of this analysis work.

The inductive approach of content analysis was used because there was a scarcity of published knowledge about intent-to-leave or intent-to-stay factors focusing on dentist leaders [19]. The results are at least suggestive and provide support for earlier studies, i.e. similar results have been obtained previously utilizing different methods and techniques. In this study most of the respondents were working in the PDS, which correspond the distribution of the dentist leaders in Finland. There are only few dentist leader positions in the private sector.

## Conclusions

Leadership and leaders have important roles today and this will increase in the future since large organizational changes are underway in many countries in all sectors of the social and health care, not only in oral health care. Therefore, it is important to know which factors support or enervate dentist leaders in their demanding posts. Education seems to be a very important factor in determining the intention to take up a leadership position as this can support work-related well-being. In this study, participants in both groups regarded working as a leader as a positive challenge and a good opportunity to supplement or compensate for clinical work. According to our results, important supporting and intent-to-stay factors, which the Stayer group mentioned, were enthusiasm for leadership, the real impact possibilities and a positive working community. As contradictory, enervating and intent-to-leave factors, which the Leaver group highlighted, were stress, the excessive number of the duties, the loneliness, and the lack of support in the leadership position as well as staff related difficulties.

Working as a dentist leader is both demanding and challenging. In order for an individual to succeed and be personally satisfied and fulfilled in this kind of leadership position, it is essential that he/she is provided with education, support, and time for leadership.
